# Health-related and cancer-related Internet use by patients treated with total laryngectomy

**DOI:** 10.1007/s00520-019-04757-6

**Published:** 2019-04-16

**Authors:** Cornelia F. van Uden-Kraan, Femke Jansen, Birgit I. Lissenberg-Witte, Simone E. J. Eerenstein, C. René Leemans, Irma M. Verdonck-de Leeuw

**Affiliations:** 1 Department of Clinical, Neuro- and Developmental Psychology, Faculty of Behavioral and Movement Sciences, Amsterdam Public Health research institute, Vrije Universiteit Amsterdam, Amsterdam, The Netherlands; 2 Cancer Center Amsterdam (CCA), Amsterdam UMC, location VUmc, Amsterdam, The Netherlands; 3 Department of Otolaryngology-Head and Neck Surgery, Cancer Center Amsterdam (CCA), Amsterdam UMC, location VUmc, Amsterdam, The Netherlands; 4 Department of Epidemiology and Biostatistics, Amsterdam UMC, location VUmc, Amsterdam, The Netherlands

**Keywords:** Quality of life, Larynx, Statistics

## Abstract

**Objectives:**

To investigate among patients treated with a total laryngectomy (TL) (1) Internet-use and Internet use to search for information on health and cancer (content); (2) which patients are most likely to use the Internet in general, for health-related and cancer-related purposes; (3) which other types of eHealth (community, communication, care) are used; and (4) preferences towards future use.

**Methods:**

Patient members of the Dutch TL patient society were asked to complete a questionnaire on Internet use, health-related and cancer-related Internet use, types of eHealth, preferences towards future use, socio-demographics, clinical factors, and quality of life (QOL). Factors associated with Internet use and health-related and cancer-related Internet use were investigated using stepwise logistic regression analysis.

**Results:**

In total, 279 TL patients participated, of whom 68% used the Internet. Of these, 63% used the Internet to search for information on health and 49% on cancer. Younger and higher educated TL patients and those with better QOL used the Internet more often. Patients with worse QOL searched more often for health-related information. Younger patients and those with shorter time since TL searched more often for cancer-related information. The current use of eHealth for communication, community, and care purposes among Internet users was limited (range, 2 to 15%). Many were interested in using these types of eHealth in the future (range, 21 to 72%).

**Conclusion:**

The majority used the Internet, especially to search for information on health and cancer, but only few for communication, community, or care purposes. Many were interested in future use.

## Introduction

Laryngeal cancer is diagnosed in around 157,000 persons worldwide each year [1]. In advanced cases, or when the disease recurs after initial treatment with (chemo)radiotherapy, treatment choice is often total laryngectomy (TL). TL is a lifesaving surgical procedure [2] and is believed to be more emotionally traumatic than any other type of surgery [3, 4]. Patients often encounter great difficulties related to altered airway, loss of voice, altered swallowing, changes in taste, loss of nasal function, difficulties in neck and shoulder movement, and social embarrassment [5, 6]. These changes have a profound effect on a patient’s post-treatment well-being and quality of life (QOL) [3, 5, 7–9]. Therefore, patients treated with TL have extensive information and supportive care needs [10].

Alongside usual care, eHealth (using information and communication technology, especially the Internet, to improve health care [11]) has the potential to facilitate these needs. Patients can be informed about their condition and the rehabilitation process via the Internet, have the opportunity to solicit medical advice from their physicians by email, and are able to share concerns with peers in online communities. In addition, self-management strategies (e.g., self-care skills education) to support patients in adopting an active role in managing their own care can be offered by means of eHealth [12, 13]. A recent meta-review on the effects of eHealth among cancer patients showed clear evidence for improvement in perceived support, knowledge levels, and information competence, and indications of evidence for improvement in health status and healthcare participation [14].

Until now, it is unknown to which extent patients treated with TL profit from eHealth. We lack insight into the potential reach of eHealth (the number, proportion, and representativeness of individuals able and willing to make use of eHealth [15]) for patients treated with TL. An important condition to make use of eHealth is to have access to the Internet. Although the percentage of persons with access to the Internet is high in the Netherlands (94%) [16], Internet access is still not complied within a relatively large group consisting of elderly (> 65 years) and chronically ill [17].

In addition, when developing eHealth applications, end users’ needs should be addressed [18, 19]. For future eHealth applications targeting TL patients to become useful and effective, it is important to have knowledge of eHealth needs and preferences of patients treated with TL.

Therefore, the aim of this study was to investigate among TL patients (1) Internet-use in general and to search for information on health and cancer (content) (2); which patients (based on socio-demographic and clinical characteristics and QOL) are most likely to use the Internet in general, for health-related and cancer-related purposes (3); which other types of eHealth (community, communication, care) are currently used; and (4) preferences towards future use of eHealth.

## Materials and methods

### Design and study population

All 914 members of the national Dutch TL patient society were asked to participate in a cross-sectional study in November 2014. They were sent a written questionnaire, consisting of items on socio-demographic variables (age, gender, living situation, education, and employment) and clinical variables (date of TL surgery and treatment with (chemo)radiotherapy), and measures on Internet use and QOL. Patients were included when they were treated with TL and were older than 18 years. According to the Dutch Medical Research Involving Human Subjects Act, ethical approval was not necessary; patients were not subjected to procedures or required to follow rules of behavior.

### Measures

#### Internet use

Internet use, health-related Internet use, and cancer-related Internet use were assessed based on an existing questionnaire on Internet use by cancer patients [20]. This questionnaire covers three broad eHealth application areas as defined by Eysenbach [21]: content (searching for health-related information), communication (soliciting medical advice from online health professionals or contact a physician or health care organization by means of the Internet), and community (sharing concerns and experiences with peers in online communities). Because of the growing interest in eHealth as a means to improve supportive care and self-help, we added items, which we refer to as care.

Patients were asked if they used the Internet. Patients who did were asked if they had ever used the Internet to search for information on health and on cancer. Subsequently, those patients who did were asked on whose initiative they searched for information about cancer (e.g., on initiative of their physician), on timing; when they searched for information during their cancer trajectory (e.g., right after being diagnosed or during treatment); and the frequency (on a four-point scale ranging from one (daily) to four (one to three times a year)). These patients were also asked which type of information they searched for (e.g., consequences of treatment) and if they had been able to trace this information. Patients were asked in which way they searched (e.g., by means of a search engine) and how often they made use of specific websites (e.g., their hospital). In addition, the questionnaire contained questions on the perceived effects of searching for information on cancer, on feeling better informed, and on frequency of physician visits. Patients were also asked if they had ever discussed the cancer-related information with a health care professional (a general practitioner (GP), physician, or oncology nurse) and if this professional appreciated this.

In addition, patients who made use of the Internet in general were asked if they currently made use of eHealth with regard to the communication area (e.g., pose a question to a health care professional via Internet), community area (e.g., participate in an online support group), and care area (e.g., participate in a self-help course). Finally, the questionnaire contained items on preferences for future use of eHealth.

#### Quality of life

QOL was measured with the EuroQol-dimensions (EQ-5D), consisting of five items measuring problems on five dimensions of quality of life (mobility, care, usual activities, pain/discomfort, and anxiety/depression). TL patients could answer that they had no problems, some problems, or extreme problems [22].

### Statistical analysis

Statistical analyses were performed using the IBM Statistical Package for the Social Science (SPSS) version 22 (IBM Corp., Armonk, NY USA). Descriptive statistics were used to summarize the socio-demographic and clinical characteristics and the data on Internet use and QOL.

We defined health-related Internet users as “TL patients who had ever searched for information about their health on the Internet” and cancer-related Internet users as “TL patients who had ever searched for information about cancer on the Internet”. Chi-squared tests, independent samples *t* tests, and Mann-Whitney tests were used to compare socio-demographic and clinical characteristics among TL patients. Statistical significance was assumed when *p* value was < .05 (two-tailed).

Stepwise logistic regression analyses were performed to assess the relative importance of the variables (Nagelkerke R^2^) that were univariately associated (*p* value to enter < .05) with Internet use, health-related Internet use, or cancer-related Internet use. In the first block of the regression analysis, the demographic characteristics were entered (age, gender, education level, and employment status), followed by the clinical characteristics and QOL in the second block (time since TL and QOL).

## Results

### Study sample

Of the 914 patients who were sent a questionnaire, 288 responded (32%). In this study, only those who completed the items on use of the Internet and the use of Internet to search for information on health and cancer (*N* = 279) were included. Characteristics of the study sample are described in Table 1.

**Table 1:** Patient characteristics by Internet use (*N* = 279)

	Total *N* = 279	Internet users *N* = 191	Non-Internet users *N* = 88	*p*	Health-related Internet use *N* = 120	Non-health-related Internet use *N* = 71	*p*	Cancer-related Internet use *N* = 94	Non-cancer-related Internet use *N* = 97	*p*
Age in years^a^				< .001^***^			.013^*^			.001^**^
Mean (SD)	70 (9.3)	68 (9.3)	73 (8.2)		67 (9.0)	71 (9.6)		66 (8.6)	71 (9.5)	
Minimum	45	45	49		47	45		48	45	
Maximum	92	90	92		90	90		90	90	
Gender^b^, *n* (%)										.014^*^
Male	233 (85)	163 (86)	70 (81)		97 (82)	66 (93)		73 (79)	90 (93)	
Female	42 (15)	26 (14)	16 (19)		21 (18)	5 (7)		19 (21)	7 (7)	
Living situation^c^, *n* (%)										
Living alone	58 (21)	35 (19)	23 (27)		22 (19)	13 (19)		18 (20)	17 (18)	
Living with partner	187 (69)	134 (72)	53 (62)		83 (71)	51 (73)		64 (70)	70 (74)	
Living with partner and children	19 (7)	15 (7)	5 (6)		8 (7)	6 (9)		6 (7)	8 (8)	
Other (e.g., in an institution)	8 (3)	4 (2)	4 (5)		4 (3)	0 (0)		4 (4)	0 (0)	
Education^d^, *n* (%)				< .001^***^						
Elementary education	27 (10)	9 (5)	18 (21)		4 (3)	5 (7)		4 (4)	5 (5)	
Lower education	131 (48)	77 (41)	54 (62)		48 (41)	29 (41)		39 (42)	38 (39)	
Secondary education	68 (25)	54 (29)	14 (21)		37 (31)	17 (24)		30 (33)	24 (25)	
Higher education	50 (18)	49 (26)	1 (1)		29 (25)	20 (28)		19 (21)	30 (31)	
Employment, *n* (%)				< .001^***^			^*.*^025^*^			^*.*^024^*^
Employed in paid work	33 (12)	30 (16)	3 (3)		21 (18)	9 (13)		16 (17)	14 (14)	
Not employed/not able to work	42 (15)	35 (18)	7 (8)		28 (23)	7 (10)		24 (26)	11 (31)	
Retired	204 (73)	126 (66)	78 (89)		71 (59)	55 (78)		54 (57)	72 (74)	
Time passed since TL in years^e^							.005^**^			< .001^***^
Median (IQR)	7 (2–14)	7 (2–14)	7 (3–15)		5 (2–5)	10 (3–10)		4 (1–10)	10 (0–34)	
Received other treatments^f^										
No	52 (19)	37 (20)	15 (18)		20 (17)	17 (24)		15 (16)	22 (23)	
Yes, radiation	199 (73)	136 (72)	63 (75)		87 (74)	49 (69)		69 (74)	67 (70)	
Yes, chemoradiation	22 (8)	16 (9)	6 (7)		11 (9)	5 (7)		9 (10)	7 (7)	
Quality of life^g^ (utility score)				.037^*^			.001^**^			.005^**^
Median (IQR)	.89 (.81–1.00)	.88 (.81–1.00)	.88 (.72–1.00)		.86 (.78–1.00)	1.00 (.84–1.00)		.85 (.78–1.00)	1.00 (.81–1.00)	

*p* for chi-squared test, independent sample *t* test, or Mann-Whitney *U* test comparing Internet users versus non-Internet users, health-related Internet users versus non-health-related Internet users, and cancer-related Internet users versus non-cancer-related Internet users

*SD* standard deviation, *IQR* interquartile range

*p < .05; **p < .01; ***p < .001

^a^Information on age is missing in 2 patients. ^b^Information on gender is missing in 4 patients. ^c^Information on living situation is missing in 7 patients. ^d^Information on education is missing in 3 patients. ^e^Information on mean number of years since total laryngectomy is missing in 14 patients. ^f^Information on other treatments besides total laryngectomy is missing in 6 patients. ^g^Information on quality of life is missing in 8 patients

### Internet use in general and use of the Internet to search for information on health and cancer

In total, 68% (*N* = 191) made use of the Internet in general. Of the Internet users 63% (*N* = 120) searched for information on health and 49% (*N* = 94) searched for information on cancer (Table 1).

Within the group of patients who used the Internet to search for information on cancer (*N* = 94), most searched on their own initiative (*N* = 79, 84%). Only a few were referred to the Internet by their physician (*N* = 6, 6%), (oncology) nurse (*N* = 8, 9%), or their GP (*N* = 1, 1%).

TL patients searched most frequently for information about cancer immediately after being diagnosed. In total, 62% (*N* = 49) searched for information about cancer daily to several times per week during this phase. During treatment with (chemo)radiation, daily-to-weekly Internet use for cancer-related purposes decreased to 42% (*N* = 27) (only relevant for those treated with (chemo)radiation). In the follow-up phase, most TL patients (*N* = 54, 68%) searched for information about cancer several times per month to one to three times a year.

Patients most often searched for information about their tumor type and (consequences) of treatment and were able to find the information they searched for (Table 2).

**Table 2:** Type of information about cancer searched for (*N* = 82–91)

	Searched for and found *N* (%)	Search for and not found *N* (%)	Not searched for *N* (%)
Information on cancer and treatment			
Type of cancer	82 (90)	–	9 (10)
Treatment	75 (84)	–	14 (16)
Treatment guidelines	42 (48)	2 (2)	43 (49)
Trials/research	17 (19)	8 (9)	63 (72)
Alternative medicine	14 (16)	5 (6)	71 (79)
Options for supportive care	18 (21)	8 (9)	60 (64)
Information on health care			
Where to find a good oncologist	12 (14)	5 (6)	71 (81)
Which hospital is best	14 (16)	7 (8)	68 (76)
Information on patient support			
Patient associations	53 (59)	3 (3)	34 (38)
Cancer support groups	49 (54)	3 (3)	38 (42)
Patient activities in region	38 (43)	6 (7)	44 (50)
Information on consequences of cancer and treatment			
Consequences of treatment in general	73 (81)	–	17 (19)
Sexual issues	20 (22)	8 (9)	62 (69)
Fatigue	24 (27)	10 (11)	54 (61)
Other symptoms experienced as a consequence of treatment	34 (42)	6 (7)	42 (51)
Lifestyle and health	36 (42)	3 (4)	47 (55)
Consequences for future parenthood	2 (2)	1 (1)	81 (96)
Other type of information searched for			
Health care insurance coverage	43 (49)	5 (6)	40 (47)
Financial consequences	20 (23)	8 (9)	59 (68)
Legislation (e.g. insurances, compensation)	37 (39)	9 (10)	42 (48)
Cancer and genetics/heritability	27 (31)	5 (6)	54 (63)
End of life	8 (9)	–	79 (91)
What I can do myself	49 (52)	3 (3)	36 (41)

The majority (*N* = 70, 76%) searched for information about cancer by means of a search engine. Websites used most were those of patient associations (94%), websites like the patient information website of the Dutch Cancer Society (72%), of health organizations (63%), and of hospitals (56%).

Most patients (*N* = 55, 60%) felt better informed after searching for information on cancer online. In total, 29 patients (32%) indicated that, although they felt better informed, also, new questions had arisen. The majority (*N* = 69, 76%) indicated that consulting the Internet did not influence the frequency of physician visits. Several (*N* = 49, 54%) had ever discussed the cancer-related information with a health care professional (respectively with a physician (*N* = 39, 75%), oncology nurse (*N* = 29, 56%), or GP (*N* = 18, 35%)). Patients were of the opinion that most health care professionals appreciated discussing this information (respectively 95% physician, 93% oncology nurse, 77% GP).

### Associations between demographic, clinical characteristics and QOL and Internet use, health-related and cancer-related Internet use

Internet use in general was significantly associated with age, education level, and QOL explaining 24% of the variance (Table 3). Younger and higher educated TL patients and those with better QOL used the Internet significantly more often. Health-related Internet use was significantly associated with QOL explaining 13% of the Consistent. Patients with worse QOL searched more often for information on health. Cancer-related Internet use was significantly associated with age and time since TL explaining 20% of the variance. Younger patients and those with shorter time since TL searched more often information on cancer.

**Table 3:** Logistic regression analysis for Internet use, health-related Internet use, and cancer-related Internet use

	Model step 1, odds ratio (95% CI)	Model step 2, odds ratio (95% CI)	Nagelkerke R^2^
Internet use			
Step 1 demographics			0.22
Age (continue)	0.95 (0.91–0.98)^b^	0.95 (0.91–0.98)^b^	
Education			
Low	Reference	Reference	
High	5.24 (2.72–10.08)^c^	5.27 (2.71–10.25)^c^	
Employment			
Not employed	Reference	Reference	
Employed	1.93 (0.51–7.32)	1.69 (0.44–6.47)	
Step 2 clinical characteristics			0.24
QOL (continue)		4.71 (1.03–21.51)^a^	
Health-related Internet use			
Step 1 demographics			0.07
Age	0.96 (0.93–1.00)^a^	0.97 (0.93–1.01)	
Gender			
Male	Reference	Reference	
Female	2.48 (0.88–7.02)	2.00 (0.69–5.84)	
Step 2 clinical characteristics			0.13
Time passed since TL (continue)		0.97 (0.93–1.02)	
QOL (continue)		0.06 (0.04–0.71)^a^	
Cancer-related Internet use			
Step 1 demographics			0.13
Age (continue)	0.94 (0.91–0.98)^b^	0.96 (0.92–1.00)^a^	
Gender			
Male	Reference	Reference	
Female	2.88 (1.12–7.41)^a^	2.58 (0.96–6.96)	
Step 2 clinical characteristics			0.20
Time passed since TL (continue)		0.94 (0.90–0.98)^b^	
QOL (continue)		0.25 (0.03–2.24)	

^a^*p* < .05, ^b^*p* < .01, ^c^*p* < .001

### Types of eHealth currently used

Among TL patients who used the Internet in general, current use of eHealth for communication, community, and care was low (Table 4). With respect to the use of eHealth for communication purposes, in total, 19 patients (10%) had made an appointment online with a health care professional or hospital, 22 patients (12%) had asked their health care professional a question online, and 28 patients (15%) had ordered medication online. When focusing on the use of eHealth for community purposes, 11 patients (6%) had at some time passively participated (reading postings) and 9 patients (5%) had actively participated (also sending postings) in an online patient support group. In total, 15 patients (8%) were in contact with peers via chat or Facebook. With respect to the use of the eHealth for care, in total, 9 patients (5%) had ever searched the Internet for options for supportive care online. Only 3 patients (2%) had participated in an online self-help course.

**Table 4:** Usage of eHealth for communication, community, and care purposes by Internet users (*N* = 180–184)

	Number	Percentage
Communication		
Make an appointment with a health care professional or organization	19	10
Pose question to my health care professional via the Internet	22	12
Order medication	28	15
Search for a health care professional review	6	3
Post a health care professional review	5	3
Community		
Read along with an online support group	11	6
Send posting to online support group	9	5
Contact via chat/Facebook	15	8
Care		
Participation in a self-help course	3	2
Search for supportive care options	9	5

### Preferences regarding future use of eHealth

Many patients who made use of the Internet (*N* = 191) were interested in using eHealth in the future (Table 5). Patients were especially interested in using eHealth to communicate with health care organizations and professionals: obtaining access to their own test results (*N* = 131; 72%) and medical record (*N* = 129; 71%), followed by requesting a prescription (*N* = 122; 69%) and making an appointment online (*N* = 114; 64%). About half of the patients would like to have contact with their own physician (*N* = 99; 56%) or nurse (*N* = 89; 50%) by email. With respect to using eHealth for community purposes, TL patients showed less interest. Only 40 (22%) were interested in contacting others online. When focusing on care, the most preferred functionality concerned obtaining a personalized overview of supportive care options (*N* = 90; 50%), followed by receiving personalized advice (*N* = 80; 45%) and monitoring symptoms online (*N* = 78; 44%). Participating in self-help courses (*N* = 37; 21%) and doing self-tests online (*N* = 39; 23%) were less popular.

**Table 5:** Preferences for future use of eHealth for communication, community, and care purposes (*N* = 174–182)

	Yes (*n*, %)	Neutral (*n*, %)	No (*n*, %)
Communication			
Access to own health record	129 (71)	25 (14)	28 (15)
Access to own test results	131 (72)	20 (11)	30 (17)
e-mail with my physician	99 (56)	31 (17)	48 (27)
e-mail with nurses	89 (50)	39 (22)	50 (28)
Request prescriptions	122 (69)	26 (15)	30 (17)
Request tests	86 (49)	35 (20)	55 (31)
Make an appointment with own physician	114 (64)	31 (18)	32 (18)
Pose question to my physician via a forum	59 (34)	28 (16)	89 (51)
Review health care professional or organization	65 (37)	46 (26)	67 (38)
Report complaints	83 (46)	42 (23)	54 (30)
Suggest ideas for improvement of treatment	89 (50)	41 (23)	49 (27)
Community			
Chat with other TL patients	40 (22)	41 (23)	99 (55)
Care			
Do self-tests	39 (23)	36 (21)	98 (57)
Receive reminders in support of the treatment	69 (39)	36 (20)	72 (41)
Monitor symptoms	78 (44)	45 (25)	54 (31)
Receive personalized advice	80 (45)	36 (20)	63 (35)
Receive a personalized overview of supportive care	90 (50)	34 (19)	55 (31)
Participate in an online self-help course	37 (21)	36 (21)	101 (58)

## Discussion

Results of this study revealed that 68% of patients treated with TL make use of the Internet. In total, respectively 63% and 49% of these TL patients used the Internet to search for information about their health and to search for information about cancer. Younger and higher educated TL patients and those with better QOL used the Internet more often. Patients with worse QOL searched more often for health-related information. Younger patients and those with shorter time since TL searched more often for cancer-related information. The current use of eHealth for communication, community, and care purposes was limited (range, 2 to 15%). However, many were interested in using these types of eHealth in the future (range, 21 to 72%).

The majority of TL patients made use of the Internet. The estimated proportion of Internet usage among TL patients was comparable with the proportion of usage among the general Dutch population of 65 years and older in 2014 of 72% [16] and with the proportion of usage of 54 to 74% found in earlier studies respectively performed between 2005 and 2012 among cancer patients [20, 23–25]. Results are, however, in contrast to the expectations of health care professionals involved in the care of head and neck cancer patients, among which TL patients, who expected that eHealth interventions might not be useful for this specific patient group, due to a lack of Internet access [26]. Current study results might encourage health care professionals to promote usage of eHealth interventions among this specific patient group, potentially offering more TL patients to benefit from eHealth.

Our study confirmed previous findings on the impact of age and education [20, 23, 25] on the level of Internet usage. A recent national monitor study among the general Dutch population concluded that the gap in Internet usage between younger and older people is decreasing. Increase in Internet use among elderly is mainly resulting from people who already used the Internet and subsequently pass 65 [16]. The education gap found in our study, is consistent with literature in which an education gap in Internet use, especially for information purposes, is described [27, 28].

TL patients who made use of the Internet specifically made use of the content application area of the Internet, by seeking information on health (63%) and cancer (49%) online. Our study showed that patients with worse QOL searched more often for information on health. This is in line with results of an earlier study conducted among Dutch cancer survivors which showed that worse QOL was associated with a higher positive attitude regarding online communication with health-care professionals and online self-care [13]. Those who suffer from a poor health might have more reason to use eHealth.

Those with shorter time since TL searched more often for information on cancer. Including all members of the Dutch national TL patient society allowed including those patients who underwent TL up to 37 years ago, in an era when Internet was not available yet. Since patients who did use the Internet to search for information on cancer most frequently did so right after being diagnosed, this potentially resulted in the lower cancer-related Internet usage of TL patients treated longer ago.

The explained variance of Internet use and health-related and cancer-related Internet use found in our study was relatively low (12–28%). A potential explanatory factor that should be studied in future research concerns cancer patients’ cognitive coping style: monitoring versus blunting [29]. Under impending medical threat, such as being confronted with a cancer diagnosis, high monitors scan for potentially threatening health information, for instance by means of the Internet, while low monitors refrain from engaging in this behavior [25, 30]. Another potential explanatory factor that should be studied in future research among TL patients specifically concerns level of communication impairment. Difficulty in speaking might dictate TL patients to seek more information on the Internet, because patients often express their informational needs to health care professionals through verbal signals [31].

In line with earlier study results, we found that searching for information on health is the most common online health-related activity [20, 32]. Results showed that patients most frequently searched for information right after being diagnosed and during treatment. The frequency of searching decreased during follow-up. These findings correspond with study results on information needs of head and neck cancer patients which are highest at diagnoses, during treatment, and during the 1- to 3-month period following treatment [33]. Possibly, patients who are longer after TL have returned to a ‘new normal’ life, affecting the frequency of searching for information on the Internet.

Only a small number of TL patients made use of eHealth for other purposes, in contrast to a high interest in future use. Patients were especially interested in online communication with health care organizations and professionals (e.g., by means of obtaining access to their own test results and medical record [20, 34]) and care (e.g., by means of monitoring symptoms and obtaining and overview of personalized supportive care options [35]). Although there is already a substantial range of eHealth available, patients appear to not know about these online services and engagement thus remains low. Healthcare providers are recommended to make eHealth better known and include eHealth in the regular healthcare processes [36]. Besides, end users and other stakeholders should be included in the development process to carefully match eHealth to patients’ needs to ensure adequate uptake [18, 19, 37]. The current limited use of eHealth applications may also be explained by the fact that using eHealth for communication, community, and care asks for a more diverse range of skills than retrieving information alone [38]. Since it is not self-evident that TL patients possess these skills, future research is needed to obtain insight into digital health literacy among TL patients.

The relatively limited interest among TL patients (21%) who made use of the Internet in future participation in a self-help course might be related to inexperience. We developed an online self-care application “In Tune without Cords (ITwC)” for TL patients. This self-care application was tested on its feasibility in a group of patients that underwent TL up to 2 years ago. Study results showed that the uptake of 73% among Internet users was good and that patients were satisfied with the application [39]. When TL patients become more familiar with and have access to such online self-care interventions, they might be more likely to use them.

A limitation of this study is its cross-sectional design, impeding the ability to draw conclusions on causality of findings. Solely including TL patients who are members of the Dutch TL patient society and the relatively low response rate may have impaired representativeness to all TL patients. The low response rate may be due to the study procedures being anonymized, and we were, therefore, not able to send reminders to non-responders. Finally, a limitation of this study is that we did not study digital health literacy skills, and level of communication impairment of TL patients included.

## Conclusion

The majority of TL patients made use of the Internet, especially to search for information on health and cancer. Younger and higher educated TL patients and those with better QOL used the Internet more often. Patients with worse QOL searched more often for health-related information. Younger patients and those with shorter time since TL searched more often for cancer-related information. The current use of eHealth for communication, community, and care purposes was limited. However, many TL patients were interested in using these types of eHealth in the future. Since study results showed that the majority of TL patients make use of the Internet and are interested in eHealth, these results might encourage health care professionals in promoting eHealth in this specific patient population, potentially offering more TL patients to benefit from eHealth.
